# Isn’t here just there without a “t” – to what extent can digital Clinical Case Discussions compensate for the absence of face-to-face teaching?

**DOI:** 10.3205/zma001392

**Published:** 2020-12-03

**Authors:** Jan M. Zottmann, Anna Horrer, Amir Chouchane, Johanna Huber, Sonja Heuser, Lica Iwaki, Christian Kowalski, Martin Gartmeier, Pascal O. Berberat, Martin R. Fischer, Marc Weidenbusch

**Affiliations:** 1 LMU Munich, University Hospital, Institute for Medical Education, Munich, Germany; 2 Technical University of Munich, School of Medicine, TUM Medical Education Center, Munich, Germany; 3 LMU Munich, University Hospital, Department of Anesthesiology, Munich, Germany; 4 LMU Munich, University Hospital, Department of Internal Medicine IV, Munich, Germany

**Keywords:** case-based learning, clinical reasoning, peer teaching, curriculum development, undergraduate medical education, digitalisation

## Abstract

**Objective:** COVID-19 challenges curriculum managers worldwide to create digital substitutes for classroom teaching. Case-based teaching formats under expert supervision can be used as a substitute for practical bedside teaching, where the focus is on teaching clinical reasoning skills.

**Methods: **For medical students of LMU and TU Munich, the interactive, case-based, and supervised teaching format of Clinical Case Discussion (CCD) was digitised and implemented as dCCD in their respective curricula. Case discussions were realised as videoconferences, led by a student moderator, and took place under the supervision of a board-certified clinician. To prevent passive participation, additional cognitive activations were implemented. Acceptance, usability, and subjective learning outcomes were assessed in dCCDs by means of a special evaluation concept.

**Results:** With regard to acceptance, students were of the opinion that they had learned effectively by participating in dCCDs (M=4.31; SD=1.37). The majority of students also stated that they would recommend the course to others (M=4.23; SD=1.62). The technical implementation of the teaching format was judged positively overall, but findings for usability were heterogeneous. Students rated their clinical reasoning skills at the end of the dCCDs (M=4.43; SD=0.66) as being significantly higher than at the beginning (M=4.33; SD=0.69), with low effect size, t(181)=-2.352, p=.020, d=0.15.

**Conclusion: **Our evaluation data shows that the dCCD format is well-accepted by students as a substitute for face-to-face teaching. In the next step, we plan to examine the extent to which participation in dCCDs leads to an increase in objectively measured clinical reasoning skills, analogous to a face-to-face CCD with on-site attendance.

## Zusammenfassung

**Zielsetzung: **COVID-19 stellt Curriculums-Verantwortliche weltweit vor die Herausforderung, digitale Ersatzangebote für Präsenzlehre zu schaffen. Fallbasierte Formate unter Supervision bieten sich als Ersatz für klinisch-praktischen Unterricht am Krankenbett an, bei dem die Vermittlung klinischer Entscheidungsfindungskompetenzen im Vordergrund steht.

**Methodik: **Für Medizinstudierende der LMU und der TU München wurde das interaktive, fallbasierte und supervidierte Lehrformat der Clinical Case Discussion (CCD) digitalisiert und als dCCD in die jeweiligen Curricula implementiert. Die Falldiskussionen wurden als Videokonferenz realisiert, von einem studentischen Moderator angeleitet und fanden unter Supervision eines Klinikers auf Facharztniveau statt. Um eine passive Teilnahme zu unterbinden, wurden kognitive Aktivierungen implementiert. Mit Hilfe eines speziellen Evaluationskonzeptes wurden Akzeptanz, Usability und subjektiver Lernerfolg bei der dCCD erfasst.

**Ergebnisse:** Hinsichtlich der Akzeptanz waren die Studierenden der Meinung, durch die Teilnahme an den dCCDs effektiv gelernt zu haben (M=4.31; SD=1.37). Eine Mehrheit gab außerdem an, den Kurs weiterempfehlen zu wollen (M=4.23; SD=1.62). Die technische Umsetzung des Lehrformats wurde insgesamt positiv beurteilt, für die Usability ergab sich ein heterogenes Bild. Studierende schätzten ihre klinischen Entscheidungsfindungskompetenzen am Ende der dCCDs (M=4.43; SD=0.66) bei geringer Effektstärke signifikant höher ein als zu Beginn (M=4.33; SD=0.69), t(181)=-2.352, p=.020, d=0.15.

**Schlussfolgerung: **Unsere Evaluationsdaten zeigen, dass die dCCD von den Studierenden als Ersatz für Präsenzlehre gut angenommen wird. In einem nächsten Schritt soll überprüft werden, inwiefern die Teilnahme an dCCDs analog zu einer CCD-Präsenzveranstaltung zu einem Zuwachs an objektiv gemessenen klinischen Entscheidungsfindungskompetenzen führt.

## 1. Digital case discussions as a substitute for classroom teaching?

COVID-19 poses a challenge to curriculum managers worldwide for creating digital substitutes for classroom teaching, including at the university level. Practical bedside teaching, which is central to medical studies, poses a particular problem since strict restrictions to hospitals were and still are in place due to the pandemic [1]. For this reason, it is currently necessary to offer digital substitute courses for bedside teaching. In addition to anamnesis and physical examination techniques, bedside teaching is primarily aimed at fostering clinical reasoning (CR) skills. Against this background, case-based formats under expert supervision are promising as a substitute [2], [3].

## 2. Clinical Case Discussions promote clinical reasoning

**Figure 1:**
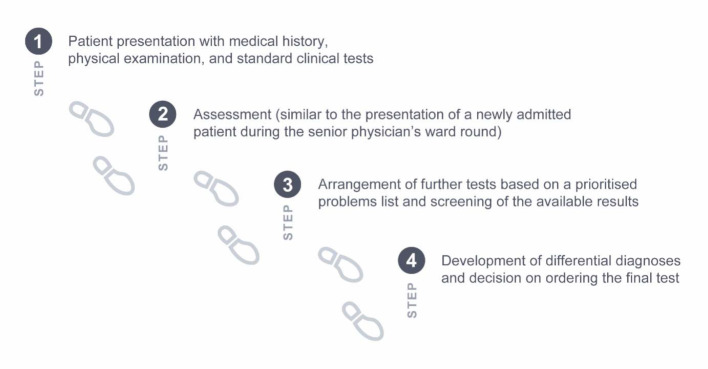
Structure of the case discussion in the CCD

For medical students at LMU and TU Munich, the teaching format of Clinical Case Discussion (CCD) has been digitised and implemented in their respective curricula. CCD is an interactive, case-based, and supervised teaching format for the promotion of CR skills [3]. Interactivity amongst its participants is encouraged by a student moderator, who is trained in didactics of higher education and who asks the fellow students questions, thus stimulating active participation in the discussion. The supervising board-certified clinician provides central learning aspects and corrects faulty CR processes. The cases discussed are derived from the New England Journal of Medicine [https://www.nejm.org/medical-articles/case-records-of-the-massachusetts-general-hospital] and are held in English according to a fixed structure (see figure 1 (Fig. 1)). A previous study found positive effects of CCDs on student CR skills, both for active participation in a face-to-face setting and for self-directed learning with video recordings of case discussions [4]. Thus, a digital variant of CCD in which students actively participate in a video conference appeared promising for the teaching of CR skills.

## 3. Digitalisation and curricular implementation of CCDs

The digitalisation of CCDs was carried out by the teaching staff of LMU and TUM, in close collaboration with the student moderators and with the support of the deans of study affairs. While the *dCCD* was offered as a compulsory elective course at TUM, it was placed in the clinical base year (6^th^ and 7^th^ semester) at LMU in which a majority of bedside teaching usually takes place. Alternatively, students could process virtual patient cases in the CASUS learning system [https://lmu.casus.net/] or embark on so-called “Corona missions” in patient care [5]. In total, about 60% of the eligible students at LMU registered for participation in dCCDs. The web conferencing software Zoom [https://zoom.us/] was used for the technical implementation at both universities.

The structure of CCDs remained essentially unchanged in the digital variant. However, additional cognitive activations were implemented to prevent lurking (i.e., passive participation) of individual participants [6]: Specifically, during step 1 of dCCD, students were required to take notes on the case presentation. After a short pause for reflection, all students sent a draft assessment to the moderator individually, which was discussed in step 2. The students also worked out the differential diagnosis dyadically or triadically in so-called "breakout rooms" (a function in Zoom where the conference can be split into separate sessions) and posted it in the chat, which in turn served as a basis for step 4.

On the basis of existing instruments [3], [7], a special CCD evaluation concept was developed that included sheets for formative and summative evaluation, as well as a sheet that could be used specifically for the implementation of this teaching format. For dCCDs, items on the acceptance or usability of the digital variant were added (see attachment 1 and attachment 2 ). Subjective learning outcomes of the students were assessed before and after the course using an established scale for the self-assessment of CR skills [8]. For the analysis, a t-test for paired samples was conducted at an alpha level of .05.

## 4. Acceptance, usability, and subjective learning outcomes of digital CCDs

Since different sheets were used for the evaluation, the number of responses available for acceptance, usability, and subjective learning outcomes varies. The final course evaluation (n=49) showed a high acceptance of the teaching format. The students felt that they had learned effectively by participating in dCCDs (M=4.31; SD=1.37). The majority of the students indicated that they would recommend this course to their fellow students (M=4.23; SD=1.62).

The technology (sound, video, presentation) of dCCDs worked reliably in the opinion of 85% of the participants (n=206); if technical problems were mentioned, these were mostly connectivity issues. With regards to the other usability items, the students were divided (see table 1 (Tab. 1)), which is probably related to the generally heterogeneous preferences of medical students regarding the use of digital learning media [9], [10] (see attachment 2 for details). With regards to subjective learning outcomes, students (n=182) judged their CR skills on a six-point Likert scale. Students rated their CR skills at the end of the course (M=4.43; SD=0.66) as being significantly higher than at the beginning of the course (M=4.33; SD=0.69), t(181)=-2.352, p=.020, d=0.15. In view of their phase of medical education, it is striking that the students’ self-assessment values were already high at the beginning of the course, suggesting that they probably overestimated their own abilities [11].

## 5. Conclusion

Our evaluation data shows that the dCCD is generally well-accepted by medical students. While the subjective learning gain was significant, it was low compared to results of a previous study of CCD in a face-to-face setting with on-site attendance [3]. We cannot completely rule out a novelty effect; on the other hand, phenomena such as “Zoom fatigue” [12] occur in association with digital teaching, which may have had a negative impact on the students’ self-assessment. In the next step, a study conducted at LMU during the 2020 summer semester will examine the extent to which participation in dCCDs leads to an increase in objectively measured CR skills. In addition, a comparison with other digital courses that were realised during the pandemic to teach clinical reasoning would be desirable.

## Funding

This work was supported by the Federal Ministry of Education and Research (grant no. 01PB18004C) and the Elite Network Bavaria (grant no. K-GS-2012-209).

## Acknowledgements

Our thanks go to the student moderators of the dCCDs at LMU and TU Munich (Julian Albers, Julia Fleig, Christine Heisen, Christopher Hemingway, Lucia Hoenen, Tilman Höing, Hugo Lanz, Charlotte Middendorf, Ekaterina Nedeoglo, Sophie Ostmeier, Aydana Rakhimbayeva, Christian Rausch, Martin Ryll, Sebastian Waldherr, Rachel Weiss, Chiara Wychera, Vladislav Yakimov), as well as to all lecturers and the team of MeCuM module 23 at LMU (Martin Dreyling, Hanna Khvorost, Mara Maticevic, Monika Merkle).

## Competing interests

The authors declare that they have no competing interests. 

## Supplementary Material

Evaluation concept for CCD

Usability of dCCD

## Figures and Tables

**Table 1:**
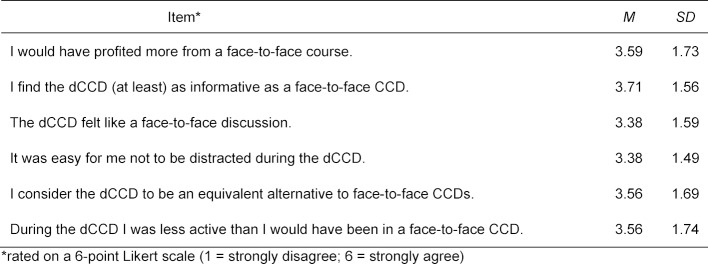
Evaluation results on the usability of the dCCD

## References

[1] Bayerisches Staatsministerium für Gesundheit und Pflege (2020). Vollzug des Infektionsschutzgesetzes (IfSG), Corona-Pandemie: Einschränkung der Besuchsrechte für Krankenhäuser, Pflege- und Behinderteneinrichtungen.

[2] Kassirer JP (2010). Teaching clinical reasoning: case-based and coached. Acad Med.

[3] Koenemann N, Lenzer B, Zottmann JM, Fischer MR, Weidenbusch M (2020). Clinical Case Discussions: A novel, supervised peer-teaching format to promote clinical reasoning in medical students. GMS J Med Educ.

[4] Weidenbusch M, Lenzer B, Sailer M, Strobel C, Kunisch R, Kiesewetter J, Fischer MR, Zottmann JM (2019). Can clinical case discussions foster clinical reasoning skills in undergraduate medical education? A randomised controlled trial. BMJ Open.

[5] Bayerisches Staatsministerium für Wissenschaft und Kunst (2020). Corona-Vorsorge: Wissenschaftsminister Sibler ruft gemeinsam mit Universitätskliniken Medizinstudenten zum freiwilligen Einsatz auf.

[6] Strijbos JW, De Laat MF (2010). Developing the role concept for computer-supported collaborative learning: An explorative synthesis. Comp Human Behav.

[7] Tolks D, Kiessling C, Wershofen B, Pudritz Y, Schunk M, Härtl A, Fischer MR, Huber J (2019). Lernen aus Fehlern anhand eines fallbasierten Curriculums im medizinischen Querschnittsbereich Gesundheitssysteme/Gesundheitsökonomie und öffentliche Gesundheitspflege. Gesundheitswes.

[8] van Gessel E, Nendaz MR, Vermeulen B, Junod A, Vu NV (2003). Development of clinical reasoning from the basic sciences to the clerkships: a longitudinal assessment of medical students' needs and self-perception after a transitional learning unit. Med Educ.

[9] Persike M, Friedrich JD (2016). Lernen mit digitalen Medien aus Studierendenperspektive. Arbeitspapier Nr. 17.

[10] Wong G, Greenhalgh T, Pawson R (2010). Internet-based medical education: A realist review of what works, for whom and in what circumstances. BMC Med Educ.

[11] Stark R, Gruber H, Renkl A, Mandl H (1998). Instructional effects in complex learning: Do objective and subjective learning outcomes converge?. Learn Instruct.

[12] Wiederhold BK (2020). Connecting through technology during the Coronavirus disease 2019 pandemic: Avoiding "Zoom Fatigue". Cyberpsychol, Behav Soc Network.

