# Supplementary material for: Isn’t here just there without a “t” – to what extent can digital Clinical Case Discussions compensate for the absence of face-to-face teaching?
Source: GMS J Med Educ. 2020 Dec 3;37(7):Doc99. doi: 10.3205/zma001392 (PMC7740012; doi:10.3205/zma001392)
Supplement: Evaluation concept for CCD [file JME-37-99-s-001.pdf]

Attachment 1: Evaluation concept for CCD

To support the new implementation of (d)CCDs at other faculties, a CCD evaluation concept was developed at LMU Munich and is described here in this supplement (and released for use). Our questionnaires can and should be adapted to the local teaching situation; however, we kindly ask for the appropriate citation of this publication when using the questionnaires in full or in part.

The present evaluation concept was developed at the University Hospital of LMU Munich specifically for the evaluation of CCDs, in a joint effort by the student organisers (moderators of CCD) and members of the working group Curricular Evaluation and Faculty Development of the Institute for Medical Education. The evaluation concept includes different questionnaires, each of which is specifically designed for certain phases of implementation. Like CCDs, the evaluation sheets are also based entirely in English. The sheets are further based on a six-point Likert scale (1 = does not apply at all; 6 = completely applies), with the additional option of 'no response'. For quick recognition of the individual questionnaires and increased user-friendliness, we recommend that the questionnaires be numbered and color-coded accordingly. For the assessment of subjective learning outcomes, we used an additional questionnaire with 10 items on clinical reasoning (van Gessel, Nendaz, Vermeulen, Junod, & Vu, 2003) as part of the summative evaluation.

- **Phase 1a:** New implementation of CCD

  Evaluation focus: general conditions and familiarity with the teaching format (e.g. an institution implements CCD for the first time and would like to know whether students are sufficiently familiar with the CCD format after one or two introductory sessions)

- **Phase 1b:** Formative evaluation of the CCD roles

  Evaluation focus: moderator, presenter, clinician (e.g. students evaluate different aspects of the performance of a new moderator, presenter, or clinician)

- **Phase 2:** Summative evaluation of the course

  Evaluation focus: reasons for participation; assessment of the performance of the moderators, presenters, and clinicians; moderation of the discussion; assessment of usefulness with regard to internationalisation; open questions about the fulfillment of dCCD expectations, suggestions for improvement, and positive aspects of CCDs (e.g. standard teaching evaluation of CCD at the end of the semester)

Evaluation sheet 1a (GREEN)

Evaluation sheet 1a (17 items) is particularly suitable in cases where CCD is newly implemented. Since neither moderators, clinicians, nor discussants have experience with the prototypical process and the specifics of CCD in this scenario, an evaluation of formal aspects is important in order to ensure process quality at this stage. This evaluation sheet helps to optimise the surrounding conditions, if necessary, before the focus of the evaluation in subsequent phases is shifted to the individual roles or the learning outcomes of the participants. Formal aspects relevant to the process include the adherence to the CCD rules of conduct (see Koenemann, Lenzer, Zottmann, Fischer, & Weidenbusch, 2020), a structured patient presentation up to the admission note, an atmosphere conducive to learning and discussion, the active participation of the students, an intermediate level of difficulty, and a subjective assessment of the participants as to how effectively participation in CCD promoted their clinical reasoning and diagnostic skills. Since CCDs are conducted in English, we further recommend to evaluate the participants' competence in the English language.

Evaluation sheet 1b (YELLOW)

Evaluation sheet 1b (21 items) is designed to formatively assess the performance of a (new) moderator, presenter, or clinician. For dCCD, we also used this sheet to evaluate student moderators who were experienced in face-to-face CCD (since they had no experience with the specific moderation of dCCDs). This evaluation sheet focuses, in particular, on the moderator's explanations and support of the clinical reasoning process as well as on the clinician's constructive feedback on the reasoning process.

Evaluation sheet 2 (BLUE)

Evaluation sheet 2 (35 items + 4 open questions) takes up various items from the two sheets described above, supplements them, and is suitable for the overall evaluation of CCDs (e.g. at the end of the semester). The focus of the summative evaluation is not only on reasons for participating in CCDs and assessing the performance of the individual roles, but also on the aspect of internationalisation (i.e., to what extent do the students feel that their participation in CCDs effectively prepared them for medical practice in English-speaking countries).

Evaluation sheet 1a GREEN Clinical Case Discussions

1. Number of clinical semesters: ____________________
2. Gender: (m / f / d)
3. Native speaker of English: (yes / no)
4. Previous CCD participation: (yes / no)

**Please choose, to which extent the following statements apply to you.**	**Does not apply at all**	**Does not apply**	**Generally does not apply**	**Generally applies**	**Applies**	**Completely applies**	**No response**
**1.** I know the "rules of conduct" of clinical case discussions (CCDs)	☐	☐	☐	☐	☐	☐	☐
**2.** The rules of conduct were followed today	☐	☐	☐	☐	☐	☐	☐
**3.** I am familiar with the structure of CCDs (e.g. Chief Complaint, HPI, PMHx, etc.)	☐	☐	☐	☐	☐	☐	☐
**4.** The structure of CCDs was applied today	☐	☐	☐	☐	☐	☐	☐
**5.** The moderator structured the CCD in a clear and helpful manner	☐	☐	☐	☐	☐	☐	☐
**6.** The learning atmosphere of the CCD was friendly and encouraging	☐	☐	☐	☐	☐	☐	☐
**7.** The peer-teaching format of the CCD was suitable for my learning	☐	☐	☐	☐	☐	☐	☐
**8.** The use of English as the predominant language did not hinder my learning	☐	☐	☐	☐	☐	☐	☐
**9.** I enjoyed participating in the CCD	☐	☐	☐	☐	☐	☐	☐
**10.** I actively participated in the CCD	☐	☐	☐	☐	☐	☐	☐
**11.** The difficulty level of the CCD was appropriate	☐	☐	☐	☐	☐	☐	☐
**12.** CCD is an effective way to foster my *clinical reasoning skills*	☐	☐	☐	☐	☐	☐	☐
**13.** CCD is an effective way to foster my *diagnostic skills*	☐	☐	☐	☐	☐	☐	☐

Thank you very much for your participation and feedback!

Attachment 1 to: Zottmann JM, Horrer A, Chouchane A, Huber J, Heuser S, Iwaki L, Kowalski C, Gartmeier M, Berberat PO, Fischer MR, Weidenbusch M. *Isn't here just there without a "t" – to what extent can digital Clinical Case Discussions compensate for the absence of face-to-face teaching?*
GMS J Med Educ. 2020;37(7):Doc99. DOI: 10.3205/zma001392

Evaluation sheet 1b YELLOW Clinical Case Discussions

1. Number of clinical semesters: ____________________
2. Gender: (m / f / d)
3. Native speaker of English: (yes / no)
4. Date of the CCD course that you attended:
5. Name of your moderator:

**Please choose, to which extent the following statements apply to you.**	**Does not apply at all**	**Does not apply**	**Generally does not apply**	**Generally applies**	**Applies**	**Completely applies**	**No response**
**1.** I know the “rules of conduct” of clinical case discussions (CCDs).	☐	☐	☐	☐	☐	☐	☐
**2.** The rules of conduct were followed today.	☐	☐	☐	☐	☐	☐	☐
**3.** I am familiar with the structure of CCDs (e.g. Chief Complaint, HPI, PMHx, etc.).	☐	☐	☐	☐	☐	☐	☐
**4.** The structure of CCDs was applied today.	☐	☐	☐	☐	☐	☐	☐
**5.** The moderator structured the CCD in a clear and helpful manner.	☐	☐	☐	☐	☐	☐	☐
**6.** The explanations of the moderator were clear and helpful.	☐	☐	☐	☐	☐	☐	☐
**7.** The moderator’s comments on the group reasoning during the CCD were helpful.	☐	☐	☐	☐	☐	☐	☐
**8.** The moderator activated my prior knowledge by his questions and cues.	☐	☐	☐	☐	☐	☐	☐
**9.** The moderator facilitated a lively discussion by all participants.	☐	☐	☐	☐	☐	☐	☐
**10.** The power point presentation of the case was adequately structured.	☐	☐	☐	☐	☐	☐	☐
**11.** The presenter chose meaningful teaching points on the case.	☐	☐	☐	☐	☐	☐	☐
**12.** The clinician gave enough space for the peer teaching process to occur.	☐	☐	☐	☐	☐	☐	☐
**13.** The clinician contributed adequately to my clinical knowledge.	☐	☐	☐	☐	☐	☐	☐
**14.** The clinician ensured a scientific standard of discussions during the CCD.	☐	☐	☐	☐	☐	☐	☐

Attachment 1 to: Zottmann JM, Horrer A, Chouchane A, Huber J, Heuser S, Iwaki L, Kowalski C, Gartmeier M, Berberat PO, Fischer MR, Weidenbusch M. *Isn't here just there without a "t" – to what extent can digital Clinical Case Discussions compensate for the absence of face-to-face teaching?*
GMS J Med Educ. 2020;37(7):Doc99. DOI: 10.3205/zma001392

**15.** The clinician motivated participants to come up with the clinical diagnoses.	☐	☐	☐	☐	☐	☐	☐
**16.** The feedback of the clinician was constructive and adequate.	☐	☐	☐	☐	☐	☐	☐

Thank you very much for your participation and feedback!

Attachment 1 to: Zottmann JM, Horrer A, Chouchane A, Huber J, Heuser S, Iwaki L, Kowalski C, Gartmeier M, Berberat PO, Fischer MR, Weidenbusch M. *Isn't here just there without a "t" – to what extent can digital Clinical Case Discussions compensate for the absence of face-to-face teaching?*
GMS J Med Educ. 2020;37(7):Doc99. DOI: 10.3205/zma001392

Evaluation sheet 2 BLUE Clinical Case Discussions

1. Number of clinical semesters: ____________________
2. Gender: (m / f / d)
3. Native speaker of English: (yes / no)
4. Previous CCD participation: (yes / no)

**Reasons for participation in the CCD**							
**Please choose, to which extent the following statements apply to you.**	**Does not apply at all**	**Does not apply**	**Generally does not apply**	**Generally applies**	**Applies**	**Completely applies**	**No response**
**1.** I participated in CCD because it was recommended to me by fellow students.	☐	☐	☐	☐	☐	☐	☐
**2.** I participated in CCD because I find the learning and teaching contents interesting.	☐	☐	☐	☐	☐	☐	☐
**3.** I participated in CCD because I can profit from the contents in my future capacity as a medical professional.	☐	☐	☐	☐	☐	☐	☐
**4.** The atmosphere was pleasant during CCD.	☐	☐	☐	☐	☐	☐	☐
**5.** Participating in CCD proved effective for me.	☐	☐	☐	☐	☐	☐	☐
**6.** I would recommend the course to a fellow student.	☐	☐	☐	☐	☐	☐	☐
**Prior knowledge and case discussions**							
**Please choose, to which extent the following statements apply to you.**	**Does not apply at all**	**Does not apply**	**Generally does not apply**	**Generally applies**	**Applies**	**Completely applies**	**No response**
**1.** I find it useful when students with different levels of prior knowledge discuss cases together.	☐	☐	☐	☐	☐	☐	☐
**2.** I experienced the case discussions as constructive.	☐	☐	☐	☐	☐	☐	☐
**3.** The discussions stimulated me to repeatedly scrutinize my hypotheses.	☐	☐	☐	☐	☐	☐	☐
**4.** By participating in CCD, I feel more at ease deducing further diagnostic steps from current results.	☐	☐	☐	☐	☐	☐	☐
**5.** By participating in CCD, I feel more at ease extracting relevant information from a case.	☐	☐	☐	☐	☐	☐	☐

**6.** By participating in CCD, I can critically evaluate my level of knowledge more easily.	☐	☐	☐	☐	☐	☐	☐
**7.** Knowledge acquired during CCD was readily shared in the group.	☐	☐	☐	☐	☐	☐	☐
**Prior knowledge and case discussions**							
**Please choose, to which extent the following statements apply to you.**	**Does not apply at all**	**Does not apply**	**Generally does not apply**	**Generally applies**	**Applies**	**Completely applies**	**No response**
**8.** At CCD, I learned how to develop differential diagnoses.	☐	☐	☐	☐	☐	☐	☐
**9.** I had the opportunity to make clinical decisions at CCD.	☐	☐	☐	☐	☐	☐	☐
**10.** CCD taught me the structure of a patient presentation.	☐	☐	☐	☐	☐	☐	☐
**Moderator, Presenter, Clinician**							
**Please choose, to which extent the following statements apply to you.**	**Does not apply at all**	**Does not apply**	**Generally does not apply**	**Generally applies**	**Applies**	**Completely applies**	**No response**
**1.** The moderators structured the CCDs clearly and usefully.	☐	☐	☐	☐	☐	☐	☐
**2.** The presentations/explanations of the moderators were easy to understand.	☐	☐	☐	☐	☐	☐	☐
**3.** The group's discussions of the case were appropriately examined by the moderators.	☐	☐	☐	☐	☐	☐	☐
**4.** My prior knowledge was activated by helpful questions and comments by the moderators.	☐	☐	☐	☐	☐	☐	☐
**5.** The moderators stimulated the discussion.	☐	☐	☐	☐	☐	☐	☐
**6.** The presenters created a clearly structured power point presentation.	☐	☐	☐	☐	☐	☐	☐
**7.** The presenters visualized the teaching points by embedding multimedia formats.	☐	☐	☐	☐	☐	☐	☐
**8.** The clinicians competently chaperoned the peer education process.	☐	☐	☐	☐	☐	☐	☐

**9.** The clinicians contributed their knowledge at the right time.	☐	☐	☐	☐	☐	☐	☐
**10.** The clinicians ensured the scientific standard of the discussions.	☐	☐	☐	☐	☐	☐	☐
**11.** The clinicians stimulated the discussants to make their own diagnoses.	☐	☐	☐	☐	☐	☐	☐
**12.** The clinicians' feedback was constructive and appropriate.	☐	☐	☐	☐	☐	☐	☐

International Aspects of CCD							
**Please choose, to which extent the following statements apply to you.**	**Does not apply at all**	**Does not apply**	**Generally does not apply**	**Generally applies**	**Applies**	**Completely applies**	**No response**
**1.** I had a keen interest in attaining medical experience (Famulatur, PJ, Residency) in an English-speaking country before I attended CCD.	☐	☐	☐	☐	☐	☐	☐
**2.** After having participated in CCD, I am more interested in attaining medical education in an English-speaking country.	☐	☐	☐	☐	☐	☐	☐
**3.** CCD effectively prepared me for important aspects of medical practice in English-speaking countries.	☐	☐	☐	☐	☐	☐	☐

General evaluation of the course:

1. Were your expectations of CCD fulfilled?
If not, what was missing in your opinion? (bullet points)

____

____

____

2. What else do you wish for at CCD for you to feel better prepared for your clinical work?

____

____

____

3. What did you like/dislike most about the student-organized format of the course and why? (bullet points)

____

____

____

4. Is there anything else you would like to mention, which was not already mentioned above?

____

____

____

Thank you very much for your participation and feedback!

Attachment 1 to: Zottmann JM, Horrer A, Chouchane A, Huber J, Heuser S, Iwaki L, Kowalski C, Gartmeier M, Berberat PO, Fischer MR, Weidenbusch M. *Isn't here just there without a "t" – to what extent can digital Clinical Case Discussions compensate for the absence of face-to-face teaching?*
GMS J Med Educ. 2020;37(7):Doc99. DOI: 10.3205/zma001392
