# Supplementary material for: Isn’t here just there without a “t” – to what extent can digital Clinical Case Discussions compensate for the absence of face-to-face teaching?
Source: GMS J Med Educ. 2020 Dec 3;37(7):Doc99. doi: 10.3205/zma001392 (PMC7740012; doi:10.3205/zma001392)
Supplement: Usability of dCCD [file JME-37-99-s-002.pdf]

Attachment 2: Usability of dCCD

Due to pandemic-related restrictions on classroom teaching, which came into force at short notice, it was necessary to design digital learning environments for students within a short amount of time. In order to ensure the functionality and user-friendliness of these environments, close monitoring is necessary. One component of this control is the evaluation by the students (e.g. in addition to structured or unstructured feedback from the teachers). For this purpose, an additional evaluation sheet was created specifically for dCCDs. This evaluation sheet focuses on the usability of dCCDs, taking into account the learning preferences of the students. In the following, results of our usability evaluation are briefly presented. The evaluation sheet we developed is approved for use, but we ask for the appropriate citation of this publication if the questionnaire is used in full or in part.

Students spent most of their learning time studying lecture slides (median 50%), followed by eLearning and exam questions (median 30% each) as well as textbooks (median 20%) (see figure A-1). About 85% of the students stated that there were no significant technical problems with dCCDs. When technical problems were reported, however, the most common problems were related to the stability or speed of the internet connection (7%) or sound problems (3%). Five students (2% of all evaluable answers) stated that a session was cancelled due to technical problems. Just over half of the students felt that they would have benefited more from a face-to-face CCD session (57%) and that the dCCD did not really feel like a face-to-face discussion (56%). While 52% of the study participants stated that they were more active in face-to-face sessions, 49% considered dCCDs to be an equivalent alternative to face-to-face CCDs (see figure A-2). When individual learning preferences were taken into account, an analysis with one-tailed t-tests for independent samples showed significant differences between students who spend less than half of their learning time on eLearning (n=128) and students who spend 50% or more of their learning time on eLearning (n=73). Students with a preference for eLearning were more likely to think that dCCDs were as instructive as a face-to-face discussions, $t(199)=1.796$, $p=.037$, $d=0.26$. At the same time these students were less likely to think that they would have benefited more from face-to-face CCDs, $t(199)=-1.879$, $p=.031$, $d=0.27$.

Attachment 2 to: Zottmann JM, Horrer A, Chouchane A, Huber J, Heuser S, Iwaki L, Kowalski C, Gartmeier M, Berberat PO, Fischer MR, Weidenbusch M. *Isn't here just there without a "t" – to what extent can digital Clinical Case Discussions compensate for the absence of face-to-face teaching?* GMS J Med Educ. 2020;37(7):Doc99. DOI: 10.3205/zma001392

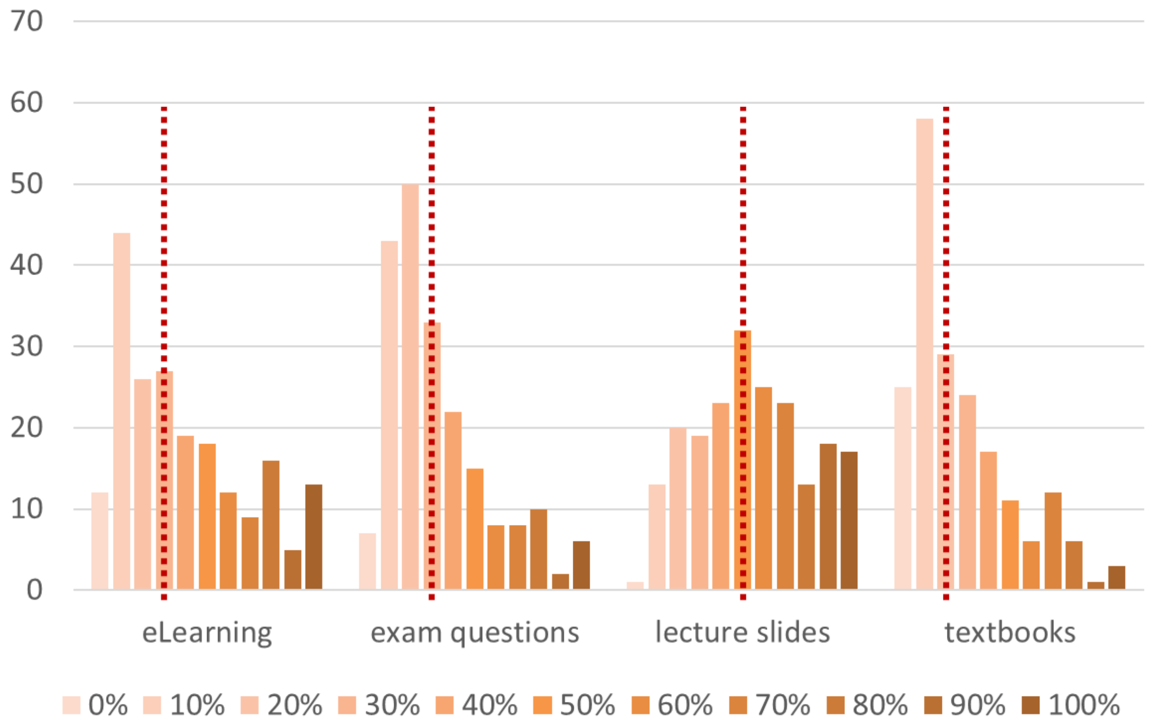

Figure A-1. Distribution of average learning time by medium. Median shown as red dotted lines (*n*=206).

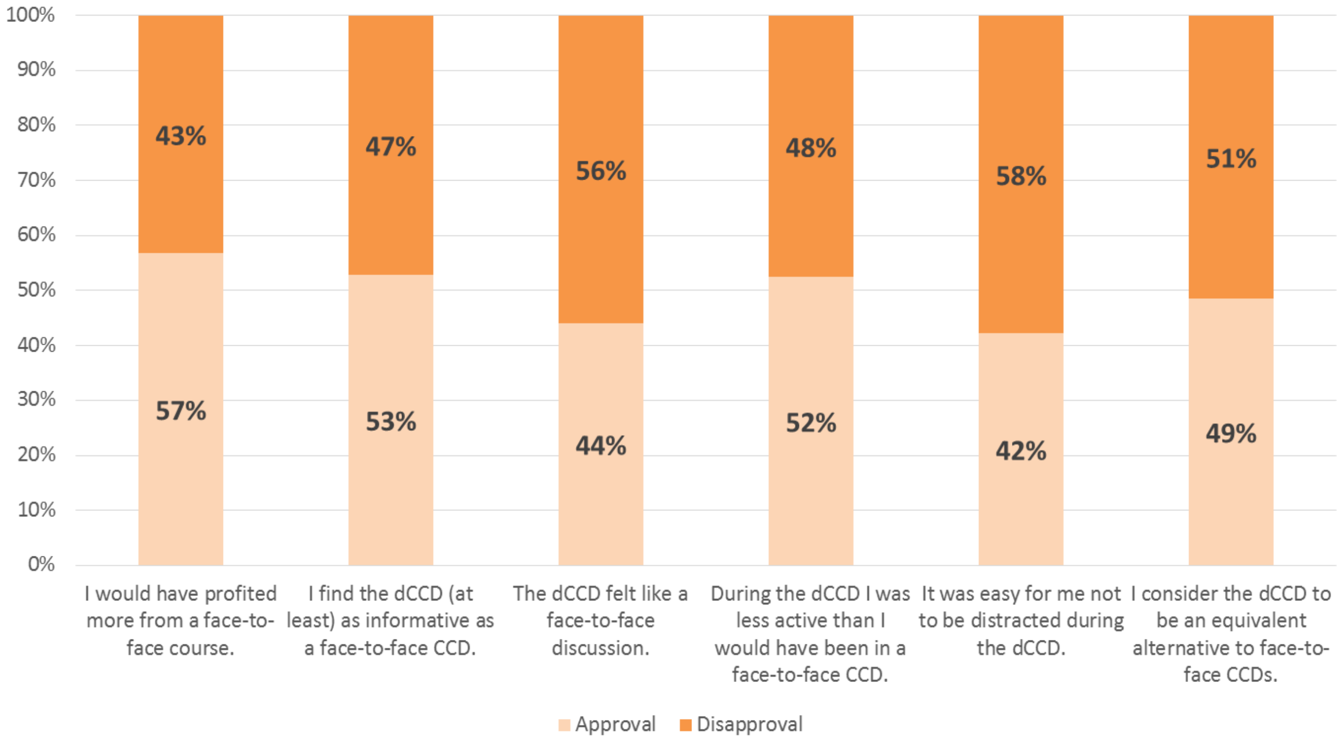

Figure A-2. Results of the usability evaluation. Representation of the six-point Likert scale, with indication of agreement/disagreement to the items in percentage (*n*=206).

Attachment 2 to: Zottmann JM, Horrer A, Chouchane A, Huber J, Heuser S, Iwaki L, Kowalski C, Gartmeier M, Berberat PO, Fischer MR, Weidenbusch M. *Isn't here just there without a "t" – to what extent can digital Clinical Case Discussions compensate for the absence of face-to-face teaching?* GMS J Med Educ. 2020;37(7):Doc99. DOI: 10.3205/zma001392

Evaluation sheet “Usability of dCCD”

Reflect upon your learning during the semester.

**What percentage of your learning time do you use**	**Percentage**	**No response**
**1.** eLearning	[Numerical value]	☐
**2.** Exam questions	[Numerical value]	☐
**3.** Lecture slides	[Numerical value]	☐
**4.** Textbooks (print and ebooks)	[Numerical value]	☐

What was your experience?

	**Yes**	**No**
**5.** Did the technology work reliably during dCCD (sound, video, presentation)?	☐	☐
**6.** If not: what technical problems did you encounter during the session?	[Free response answer]	

**Please choose, to which extent the following statements apply to you.**	**Does not apply at all**	**Does not apply**	**Generally does not apply**	**Generally applies**	**Applies**	**Completely applies**	**No response**
**7.** I would have profited more from a face-to-face course.	☐	☐	☐	☐	☐	☐	☐
**8.** I find the dCCD (at least) as informative as a face-to-face CCD.	☐	☐	☐	☐	☐	☐	☐
**9.** The dCCD felt like a face-to-face discussion.	☐	☐	☐	☐	☐	☐	☐
**10.** During the dCCD I was less active than I would have been in a face-to-face CCD.	☐	☐	☐	☐	☐	☐	☐
**11.** It was easy for me not to be distracted during the dCCD.	☐	☐	☐	☐	☐	☐	☐
**12.** I consider the dCCD to be an equivalent alternative to face-to-face CCDs.	☐	☐	☐	☐	☐	☐	☐

Thank you very much for your participation and feedback!

Attachment 2 to: Zottmann JM, Horrer A, Chouchane A, Huber J, Heuser S, Iwaki L, Kowalski C, Gartmeier M, Berberat PO, Fischer MR, Weidenbusch M. *Isn't here just there without a "t" – to what extent can digital Clinical Case Discussions compensate for the absence of face-to-face teaching?*
GMS J Med Educ. 2020;37(7):Doc99. DOI: 10.3205/zma001392
